# Per-Oral Endoscopic Myotomy-Induced Gastroesophageal Reflux Disease and Review of the Efficacy of Proton Pump Inhibitors as a Management Strategy: Review of the Literature

**DOI:** 10.7759/cureus.50324

**Published:** 2023-12-11

**Authors:** Talwinder K Nagi, Zoilo K Suarez, Muhammad Adnan Haider, Shaniah S Holder, Charles Vallejo, Sandipkumar S Chaudhari

**Affiliations:** 1 Internal Medicine, Florida Atlantic University Charles E. Schmidt College of Medicine, Boca Raton, USA; 2 Medicine, American University of Barbados School of Medicine, Bridgetown, BRB; 3 Cardiothoracic Surgery, University of Alabama at Birmingham, Birmingham, USA; 4 Family Medicine, University of North Dakota School of Medicine and Health Sciences, Grand Forks, USA

**Keywords:** laparoscopic heller myotomy, transoral incisionless fundoplication, gastroesophageal reflux disease (gerd), esophageal motility disorders, per-oral endoscopic myotomy

## Abstract

Per-oral endoscopic myotomy (POEM) is a minimally invasive procedure that is very effective in the treatment of achalasia, a rare esophageal motility disorder. POEM has become the first-line treatment for achalasia, with high success rates reported in the literature. However, a known complication of POEM is gastroesophageal reflux disease (GERD). The exact cause and risk factors of post-POEM GERD are not fully understood; however, a number of factors have played a role in its development. The management of post-POEM GERD is mainly by conservative measures, such as lifestyle changes and medications, like proton pump inhibitors (PPI), which are often the first-line method of treatment. However, surgical procedures, such as fundoplication, may be necessary in some patients. This literature review will discuss the effectiveness of the use of PPIs as a management strategy for post-POEM GERD, the factors that lead to PPI-resistant GERD, and other management strategies utilized in these cases.

## Introduction and background

Per-oral endoscopic myotomy (POEM) is a minimally invasive procedure that was first introduced by Ortega JA in 1980 and has been used to treat disorders of peristalsis within the gastrointestinal (GI) tract, such as achalasia, diffuse esophageal spasms (DES), gastroparesis, and, in some cases, adult Hirschsprung’s disease [1]. Achalasia is characterized by the degeneration of the myenteric plexus ganglion cells, leading to loss of peristalsis in the lower esophagus and failure of lower esophageal sphincter relaxation [1]. Patients may experience dysphagia, chest discomfort, and regurgitation of food particles [2]. Treatment of symptomatic achalasia includes medical therapy with botulinum toxin injection and oral nitrates or surgical methods that lead to symptomatic resolution, including pneumatic dilation, laparoscopic heller myotomy (LHM), and its endoscopic equivalent, POEM [2].

POEM and LHM are utilized for treatment-resistant achalasia, and although both are comparably effective in the resolution of symptoms with a success rate of 90%, studies show that POEM is more cost-effective and associated with less postoperative pain than its laparoscopic counterpart [3,4]. In a study by Ujiki et al., 18 patients underwent POEM and 21 underwent LHM. Operative time, myotomy length, Eckardt score improvement, and complication rates were equivalent; however, postoperative pain and analgesic use were higher in the LHM class. Return to activity was also significantly faster in the POEM group [4]. The clinical success of POEM is defined as a reduction of LES pressure by 50% or an Eckardt score of three or lower [1,5]. Chen et al. found that 26 out of 27 patients who underwent POEM had a significant decrease in Eckardt score from a mean of 8.3 to less than 0.7 and in the mean LES pressure from 31.6 mmHg to 12.9 mmHg [5].

Although POEM is an effective treatment, it is associated with adverse effects, including but not limited to mucosal tears, bleeding, pneumoperitoneum or pneumothorax, and gastroesophageal reflux disease (GERD) [1]. In a study of 58 patients by Teh et al., the incidence of GERD post-POEM was 43% on symptom score, 60% on endoscopy, and 56% on pH test [6]. A study by Constantini et al. discovered that GERD is more common post-POEM than post-LHM [7]. pH monitoring showed abnormal acid exposure in 38.4% of POEM patients compared to 17.1% of LHM patients, and endoscopy revealed esophagitis in 37.4% of POEM patients and 15.2% of LHM patients [7]. The management of GERD post-POEM includes the use of proton pump inhibitors (PPIs), such as omeprazole or pantoprazole [8]. However, not all patients respond to this method of management and require treatment escalation with endoscopic or surgical fundoplication [8]. This review will give a background on the POEM process and the pathophysiology and risk factors of post-POEM GERD and will discuss the use of PPIs and its efficiency as a management strategy.

## Review

The POEM procedure consists of four steps: (i) mucosotomy, which is performed 10 to 15 centimeters proximal to the gastroesophageal junction (GEJ); (ii) submucosal tunneling, which is extended distal to the GEJ to ensure complete disruption of the LES and expands the space between the muscularis propria and mucosa; (iii) myotomy proper with preservation of the outer longitudinal muscular layer; and (iv) closure of the mucosal defect with hemostatic clips or an endoluminal suturing device [3]. This procedure treats achalasia by decreasing the LES resting pressure, allowing the passage of the ingested material [1]. The LES is a smooth muscle segment that forms the esophagogastric junction (EGJ) barrier with the crural diaphragm preventing the retrograde movement of acidic gastric contents [9]. In healthy persons, the LES remains tonically contracted and transiently relaxes in response to a meal to facilitate food passage into the stomach [9]. In patients who undergo POEM, the LES resting pressure may become too low in some cases, resulting in the reflux of acid into the esophagus.

GERD may present as non-erosive reflux disease or erosive esophagitis, and symptoms include chest pain or heartburn due to acid reflux, dysphagia, persistent dry cough, and hoarseness [9]. A study by Abu-Nuwar et al. explored the independent predictors for developing GERD post-POEM [10]. They included (i) normal preoperative diameter of the esophagus, (ii) LES preoperative pressure less than 45 mmHg, and (iii) obesity [10]. Reflux of the acidic gastric juices leads to esophageal epithelial cell damage, resulting in an inflammatory response with neutrophilic infiltration and a proliferative response with basal cell and papillary hyperplasia [11]. If left untreated, esophagitis and Barrett’s esophagus can occur. Esophagitis is characterized by extensive erosion, ulcerations, and, in extreme cases, stricture formation and upper GI bleeding. Chronic inflammation can lead to intestinal metaplasia of the esophageal epithelium; this is known as Barrett's esophagus [11,12]. Over time, this can lead to esophageal adenocarcinoma, so long-term postoperative surveillance and early management are required to avoid such detrimental complications [11,12].

Since POEM-derived GERD has the same pathophysiology and effects as other causes of GERD, the main treatment utilized is PPIs [8]. PPIs decrease acid secretion in the stomach by blocking the H+/K+ ATPase enzyme, also known as the proton pump within parietal cells [11]. This decreases the amount of chemical damage inflicted on the esophageal mucosa. According to numerous studies, due to their consistent acid suppression, PPIs are considered the most effective medical therapy for GERD regardless of its underlying etiology [12]. Table 1 summarizes the various reports of PPI use in the management of POEM-induced GERD, highlighting the incidence of POEM-induced GERD cases, the efficacy of PPIs in these cases, and whether escalation in treatment was required [13-20]. In addition to the cases mentioned, this review aims to increase physician awareness about the association between the POEM procedure and the development of GERD and the management strategies that may be used to treat this.

**Table 1:** Incidence of GERD post-POEM, management strategies used, and whether symptomatic resolution occurred with medical or surgical modes of treatment. POEM: peroral endoscopic myotomy, PPI: proton pump inhibitor, BID: twice a day, N: number of patients

Author	Year	POEM patient sample	Number of patients with reflux symptoms or esophagitis	PPI use	Symptom resolution	Fundoplication	Symptom resolution
Nabi et al. [13]	2020	209	167	Yes (N=167)	Complete in 81.4%	-	-
Tyberg et al. [14]	2018	5	5	Yes (N=5)	A mixture of complete or partial control	Yes (N=5)	100%
Shiwaku et al. [15]	2015	2905	1886	Yes (N=1886)	Symptomatic control in 100% at 5-year follow-up	-	-
Brewer Gutierrez et al. [16]	2019	9	9	Yes (N=9)	No full resolution	9	66.67% with decreased PPI usage in all patients
Khashab et al. [17]	2016	60	6	Yes (N=6)	Complete in 100%	-	-
Chavez et al. [18]	2016	51-year-old female	Symptomatic reflux with Grade C esophagitis	Yes (PPI BID)	Partial improvement with Grade B esophagitis	Yes	Complete
Brewer Gutierrez et al. [19]	2020	146	67	Daily (N=48) Occasionally (N=15)	Complete resolution in 100%	-	-
Perbtani et al. [20]	2019	233	72	Yes (N=72)	Complete resolution (N=63) (87.5 %) Refractory (N=9)	Yes in 5 patients	Complete in 100%

According to the literature, monotherapy with PPIs is effective in the management of GERD post-POEM and the need for fundoplication is relatively low. Most cases resolve at least partially or completely with only a few instances of having to escalate treatment to fundoplication. Shiwaku et al. found that PPIs were more effective for reflux esophagitis at the five-year follow-up than those after one year [15]. Although PPIs are effective for GERD post-POEM, the indications for PPI use are not well defined due to the discrepancy between the clinical symptoms and objective assessment of esophagitis with 24-hour pH monitoring and endoscopy. Inuone et al. found that in post-POEM patients, the rate of pH positivity and findings of esophagitis on endoscopy were higher than symptomatic reflux, so it is uncertain whether PPIs are given for symptomatic relief or for healing the esophagitis [21]. 

Post-POEM GERD refractory to medical management is treated with fundoplication. Predisposing factors for PPI-resistant GERD are not well known, but some studies suggest that there is an underlying genetic role. PPI efficacy can be affected by the genotype CYP2C19, specifically rapid or intermediate metabolizers. Perbtani et al. found that eight out of nine patients who had refractory GERD despite being on PPI BID were rapid or intermediate metabolizers on CYP2C19 genotype testing [20]. Treatment in these cases entailed switching the PPI to rabeprazole since it was less affected by CYP2C19 or performing a fundoplication, both of which led to complete symptom control [20]. Using transoral incisionless fundoplication (TIF) as an alternative treatment method to PPI or in the cases of refractory GERD has its benefits. In a study by Tyberg et al., all patients who underwent TIF were able to discontinue PPI use with healing of the esophagitis that was present on esophagogastroduodenoscopy (EGD) [14]. They suggest that TIF is a safe and efficacious alternative in improving reflux symptoms, decreasing long-term complications of acid exposure, and decreasing the risks of long-term PPI use [14].

## Conclusions

POEM is a revolutionary procedure that is safe, efficient, and associated with minimal complications. GERD is a complication that affects many patients after the POEM procedure, which can lead to continued discomfort and the risk of many severe and detrimental health consequences. Prolonged postoperative monitoring allows for early detection and management of symptoms. Treatment with PPI is efficient and cost-effective and leads to complete resolution in many cases, but some cases of GERD may be refractory, leading to the need for alternative therapies, such as fundoplication. TIF has been found to improve patients’ symptoms in small case reports and cohorts, but a larger cohort sample size is required to confirm its benefit. Selecting the proper treatment modality for post-POEM GERD is dependent on the patient’s preferences and should be uniquely tailored to benefit the patient without affecting their quality of life.
